# Stigma and self-stigma in patients with delusional disorder: a systematic review.

**DOI:** 10.1192/j.eurpsy.2023.2214

**Published:** 2023-07-19

**Authors:** A. González- Rodríguez, A. Alvarez, E. Román, A. Guàrdia, M. Natividad, M. Alberto Marcus, E. Calvo, J. Labad, J. A. Monreal

**Affiliations:** 1 Mental Health, Mutua Terrassa University Hospital. University of Barcelona (UB). CIBERSAM, Terrassa; 2 Mental Health, Hospital of Mataró. I3PT. CIBERSAM, Mataró; 3 Mental Health, Mutua Terrassa University Hospital. University of Barcelona (UB). Inst. Neurosc. UAB. CIBERSAM, Terrassa, Spain

## Abstract

**Introduction:**

The association between insight, stigma and self-concept has been considered as a potential predictor of poor clinical outcomes and global functioning in psychosis. In patients with delusional disorder (DD), the effects of stigma and self-stigma have been poorly explored.

**Objectives:**

Our main goal was to systematically review studies addressing stigma and self-stigma in DD to assess whether these phenomena have an impact on clinical symptoms.

**Methods:**

A systematic review was conducted through PubMed and Google Scholar databases from inception to 2022 (PRISMA guidelines). Search terms: (Stigma OR self-stigma) AND (“delusional disorder” OR psychosis OR paranoia). Studies were considered eligible if they included patients with DD.

**Results:**

A total of 875 records were retrieved, from which 18 were included.

*Stigma*: (1) Stigma is associated with poor quality of life, poor adherence to medications and acceptation of diagnosis. (2) Support at workplaces would improve stigma and discrimination in DD. (3) Poor interpersonal competence may increase stigma experience in DD.

*Self-stigma*: (1) Women show higher level of self-stigma than men. (2) Higher rates of psychiatric hospitalizations and higher severity of symptoms associated with greater degree of self-stigma. (3) Suicidal ideation was associated with negative self-schema but not self-stigma, particularly in patients with persecutory delusions. (4) Self-stigmatization negatively associated with quality of life. (5) Depressive symptoms associated with higher levels of self-stigma. (6) Promotion interventions should address self-stigma content.

**Conclusions:**

Further longitudinal studies are needed to test the influence of stigma and self-stigma on adherence to follow-up and specific interventions to improve them.

**Disclosure of Interest:**

None Declared

